# Molecular and Electronic Structures of Macrocyclic Compounds Formed at Template Synthesis in the M(II)—Thiocarbohydrazide—Diacetyl Triple Systems: A Quantum-Chemical Analysis by DFT Methods

**DOI:** 10.3390/molecules28114383

**Published:** 2023-05-27

**Authors:** Oleg V. Mikhailov, Denis V. Chachkov

**Affiliations:** 1 Department of Analytical Chemistry, Certificatioin and Quality Management, Kazan National Research Technological University, K. Marx Street 68, 420015 Kazan, Russia; 2 Kazan Department of Joint Supercomputer Center of Russian Academy of Sciences—Branch of Federal Scientific Center “Scientific Research Institute for System Analysis of the RAS”, Lobachevskii Street 2/31, 420111 Kazan, Russia; de2005c@gmail.com

**Keywords:** template synthesis, Ni(II), Cu(II), Zn(II), thiocarbohydrazide, diacetyl, 3,10-dithio-6,7,13,14-tetramethyl-1,2,4,5,8,9,11,12-octaazacyclotetradecatetraene-1,5,7,12, DFT method

## Abstract

Using density functional theory (DFT) B3PW91/TZVP, M06/TZVP, and OPBE/TZVP chemistry models and the Gaussian09 program, a quantum-chemical calculation of geometric and thermodynamic parameters of Ni(II), Cu(II), and Zn(II) macrotetracyclic chelates, with (NNNN)-coordination of ligand donor centers arising during template synthesis between the indicated ions of *3d* elements, thiocarbohydrazide H_2_N–HN–C(=S)–NH–NH_2_ and diacetyl Me–C(=O)–C(=O)–Me, in gelatin-immobilized matrix implants was performed. The key bond lengths and bond angles in these coordination compounds are provided, and it is noted that in all these complexes the MN_4_ chelate sites, the grouping of N_4_ atoms bonded to the M atom, and the five-membered and six-membered metal chelate rings are practically coplanar. NBO analysis of these compounds was carried out, on the basis of which it was shown that all these complexes, in full accordance with theoretical expectations, are low-spin complexes. The standard thermodynamic characteristics of the template reactions for the formation of the above complexes are also presented. Good agreement between the data obtained using the above DFT levels is noted.

## 1. Introduction

**Figure 1:**
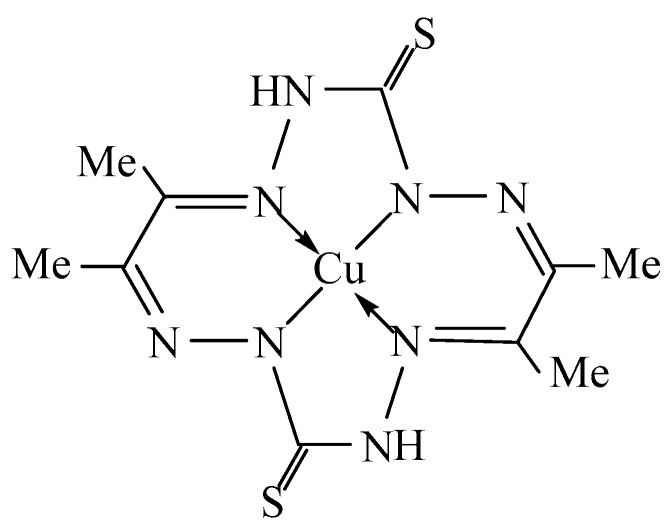
The structural formula of the complex formed during template synthesis in the Cu(II)–thiocarbohydrazide–diacetyl system as described in [1].

**Figure 2:**
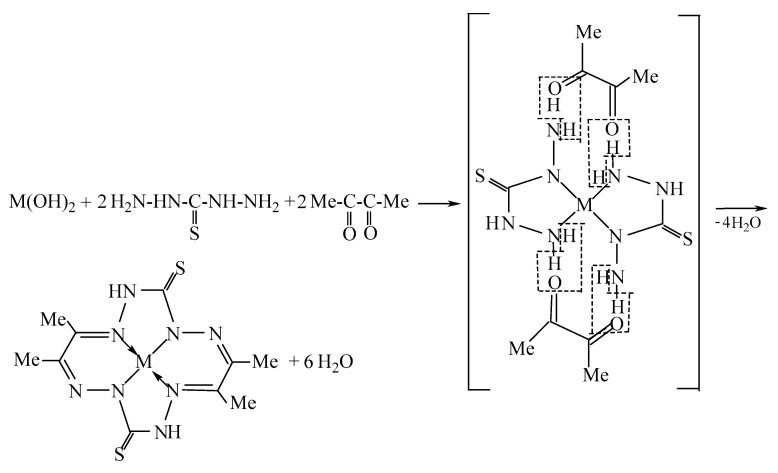
General scheme of the key reaction of template synthesis in the M(II)–thiocarbohydrazide–diacetyl systems (M = Ni, Cu, Zns).

Previously, in [1], the template synthesis in the system Cu(II)–thiocarbohydrazide H_2_N–HN–C(=S)–NH–NH_2_–diacetyl Me–C(=O)–C(=O)–Me in copper(II)hexacyanoferrate (II) gelatin-immobilized matrix implants and the formation of a macrotetracyclic chelate of the indicated metal ion were described, in which the ligand contained in it, namely, the double-deprotonated form 3,10-dithio-6,7,13,14-tetramethyl-1,2,4,5,8,9,11,12-octaazacyclo-tetradecatetraene-1,5,7,12 (**L**^2−^), coordinated to Cu(II) via four donor nitrogen atoms (Figure 1). The question of the specificity of the molecular structure of this complex (Cu**L**), however, has not yet been resolved, because neither in [1] nor any other researchers have been able to obtain single crystals of this compound suitable for XRD analysis. The complexes considered in the article [1] belong to the category of metal-macrocyclic compounds with a closed loop, in which the complex metal ion is located in the internal cavity of the macrocyclic organic compound. Such complexes have a number of unique properties that are not inherent in the complexes of the same metals with acyclic ligands and, for this reason alone, are of considerable interest to modern fundamental chemistry. On the other hand, these complexes and the macrocyclic ligands contained in them are quite close in their structure to metal-porphyrins and metal-porphyrazines (complexes formed by porphyrin and porphyrazine, respectively) and can be considered as their peculiar “precursors”. The field of the practical application of metal-porphyrins and metal-porphyrazines is very significant (optics, luminescence, catalysis, medicine, sensorics, etc.), as a result of which this group of coordination compounds is one of the most studied at present. In this context, it seems interesting and important to obtain objective data on the structural and geometric parameters of this chemical compound using quantum-chemical calculations using some version of the density functional theory (DFT), which is currently the most popular method for calculating the molecular and electronic structures of 3*d*-element complexes. On the other hand, it is very likely that in a similar way, i.e., as a result of the combination of the above ligand synthons, accompanied by intramolecular dehydration (Figure 2), the same complexes of chemical elements adjacent to copper, nickel, and zinc can be formed, although there is no information about this possibility either in [1] or in any other publications devoted to template synthesis. Since it is these two 3*d* elements that, in terms of the complexes they form, are closest to Cu(II) complexes compared to those for the other *d*-elements, it seems appropriate, first, to confirm the possibility of the existence of Ni(II) and Zn(II) metal chelates with the above ligand **L**^2−^, and secondly, in case of a positive answer to this question, compare the parameters of their molecular and electronic structures, as well as their thermodynamic characteristics, with similar parameters for the Cu**L** complex. The presentation of these data and their discussion will be carried out further in the given article.

## 2. Results

We immediately note two circumstances that are of particular importance for our further narration. First, each of the three different chemistry models we used, namely, B3PW91/TZVP, M06/TZVP, and OPBE/TZVP, unambiguously predicts the possibility of the existence of each of the three Ni**L**, Cu**L**, and Zn**L** complexes mentioned above. Second, for each of these coordination compounds, all these three methods give practically the same results, both qualitatively and quantitatively.

The key parameters of the molecular structures calculated by the three different DFT methods mentioned above, namely, the bond lengths and the angles between the lines of these bonds (bond angles) for each of the three M**L**-type chelates (M = Ni, Cu, Zn) considered by us with the above “template” ligand 3,10-dithio-6,7,13,14-tetramethyl-1,2,4,5,8,9,11,12-octaazacyclotetradecatetraene-1,5,7,12, are presented in Table 1. All three chemistry models unambiguously predict the coplanar coordination of donor nitrogen atoms of this macrocyclic ligand with respect to the central metal ion, since in the MN_4_ chelate nodes for the indicated M, the sum of four bond angles (N1M1N2), (N2M1N3), (N3M1N4), and (N4M1N1), formed by donor atoms and M atoms (**BAS**), is exactly 360.0° (which corresponds exactly to a flat quadrilateral). The same situation also takes place for the sum of non-bonding angles (NBAS) formed by neighboring donor nitrogen atoms (Table 1); consequently, the grouping of N_4_ donor atoms is also coplanar. Wherein, in none of the M**L** (M = Ni, Cu, Zn) complexes that we considered, it is rectangular—in each of them, only pairwise equality of angles (NNN) takes place, and all these angles, of course, are not equal to 90°. This difference, as expected, is most pronounced in the case of Zn**L** and least strongly in the case of Ni**L** (Table 1). As for the M–N bond lengths, taking into account the fact that they are equivalent to each other only in pairs, one should expect them to be only pairwise equal, and the calculation results for each of the three variants of the DFT chemistry models used in the work are in full agreement with this prediction. On the other hand, in the series Ni(II)–Cu(II)–Zn(II), there is an increase in ionic radii, as a result of which one can theoretically expect an increase in the lengths of these bonds; this conclusion is also in good agreement with the data presented in Table 1. Unlike the metal–nitrogen bonds, the lengths of the carbon–carbon, carbon–nitrogen, and nitrogen–nitrogen bonds show a much weaker dependence on the nature of the 3*d*-element M, but pairwise equality was also observed. The carbon–sulfur bond lengths in each of these complexes are the same, which also seems quite natural.

**Figure 3:**
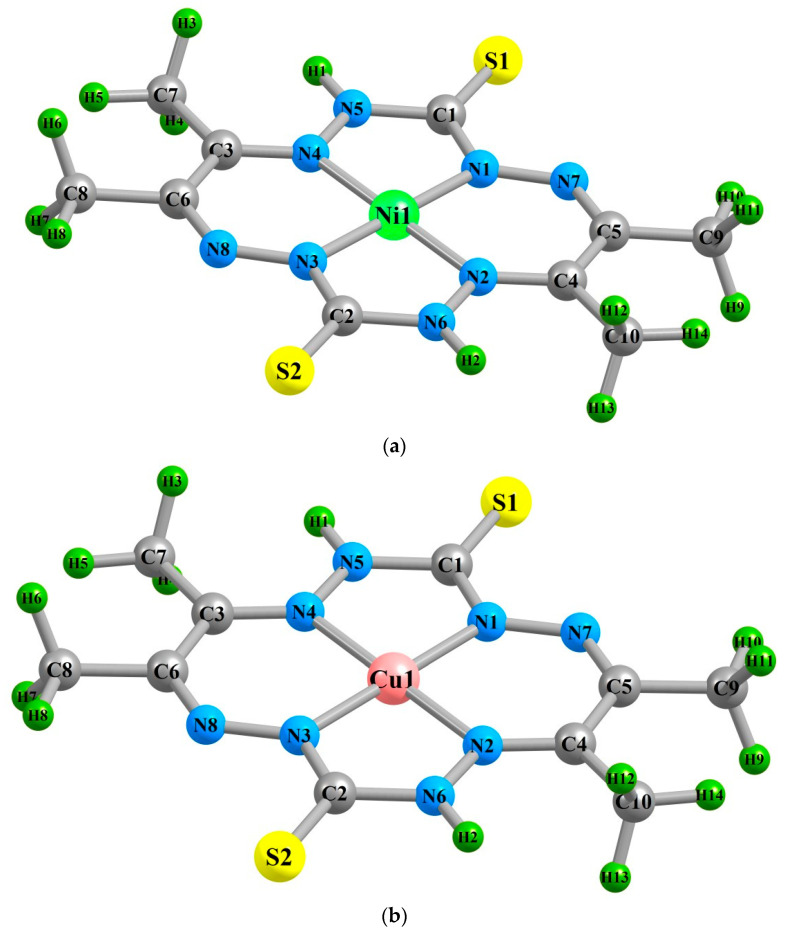

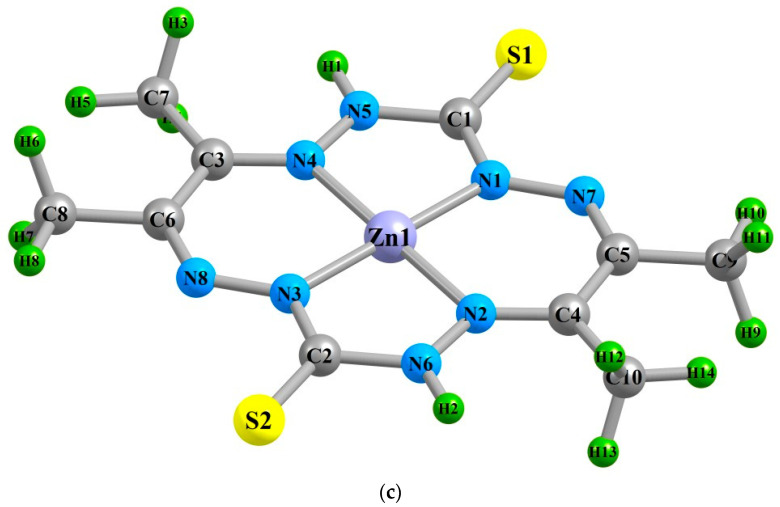
Molecular structure images of Ni**L** (**a**), Cu**L** (**b**), and Zn**L** (**c**) metal chelates calculated using the DFT B3PW91/TZVP chemistry model.

With respect to five-membered [(M1N4N5C1N1), (M1N2N6C2N3)] and six-membered [(M1N1N7C5C4N2), (M1N3N8C6C3N4)] metal chelate rings, it should be noted that all of them, as well as the MN_4_ chelate nodes, are also coplanar, since the sums of internal bond angles in any of them are either equal to 540° and 720°, which coincides with the sum of the internal angles in a flat pentagon and hexagon, respectively, or very slightly differs from these values (as is the case for the Zn**L** complex calculated by the DFT M06/TZVP method). Characteristically, these metal chelate rings are pairwise identical to each other, not only in terms of the sum of bond angles but also in their sets, which depend relatively little on the nature of M (Table 1). In view of the foregoing, the M**L** complexes under consideration can be considered practically planar (although, given that they also include, among other things, four methyl groups that are not a priori planar, it should be recognized that none of them). This fact is very interesting; considering that, according to quite numerous data presented in [2,3,4,5,6,7,8,9,10], macrotetracyclic chelate complexes of *3d* elements with cyclic ligands containing two five-membered and two six-membered metal chelate rings, contrary to theoretical expectations, are non-coplanar, and in them, as a rule, all these four cycles are non-coplanar. Each of the Ni**L**, Cu**L**, and Zn**L** complexes under consideration has one second-order axis, a horizontal plane of symmetry, and a center of symmetry, and, consequently, they all have the *C_2h_* symmetry group. In this circumstance, it is quite understandable that according to the data of our calculation by the DFT B3PW91/TZVP as well as and by the DFT M06/TZVP and DFT OPBE/TZVP, the dipole electric moment (μ) of each of them practically does not differ from zero. Images of the molecular structures of these metal chelates are presented in Figure 3.

**Figure 4:**
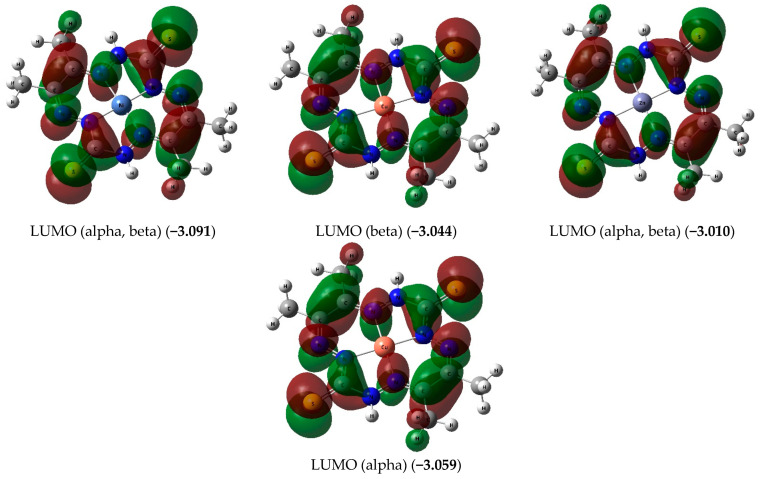

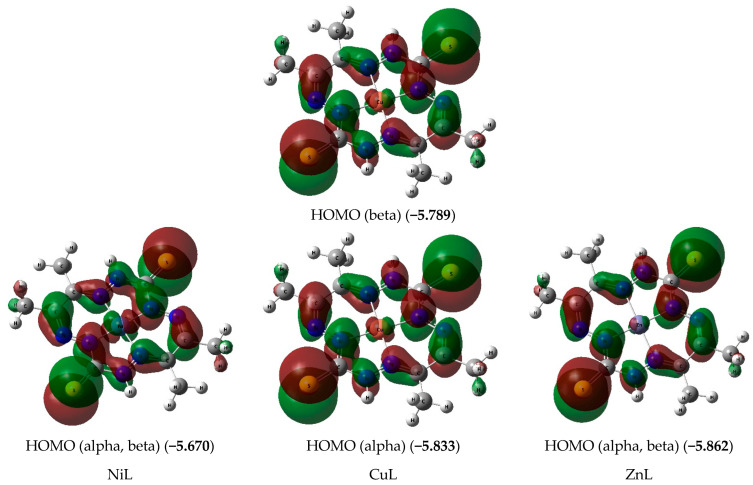
The pictures of HOMO and LUMO in the Ni**L**, Cu**L**, and Zn**L** complexes according to the DFT B3PW91/TZVP chemistry model. The energy values of the given MOs (in brackets) are expressed in eV. The symbol “alpha” belongs to the electron having spin (+1/2), and the symbol “beta” belongs to the electron having spin (−l/2).

The key data of the NBO analysis of these compounds, namely, the effective charges on the metal atoms M1 and donor nitrogen atoms N1, N2, N3, and N4 obtained by DFT B3PW91/TZVP, DFT M06/TZVP, and DFT OPBE/TZVP chemistry models, are presented in Table 2; the full NBO analysis can be found in the Supplementary Materials. As expected, they differ quite significantly from those that would take place if all bonds between atoms were ionic. This circumstance indicates a very pronounced delocalization of the electron density within the entire molecular structure of each of the complexes under consideration. Images of higher occupied (HOMO) and lower vacant (LUMO) molecular orbitals for the considered complexes are shown in Figure 4. It should be noted that the NBO analysis data for all three Ni**L**, Cu**L**, and Zn**L** complexes obtained using the above DFT variants also agree quite well with each other (Table 2).

The values of the key thermodynamic parameters of the metal chelates considered here, namely, the standard enthalpies, entropies, and Gibbs energies of their formation Δ*_f_H*^0^_298_, *S_f_*^0^_298_, Δ*_f_G*^0^_298_, are presented in Table 3. As can be seen from it, the values of *S_f_*^0^_298_ obtained by different versions of the DFT are quite close to each other, while the values of Δ*_f_H*^0^_298_ and Δ*_f_G*^0^_298_ differ quite significantly from each other. Currently, it is not possible to give preference to any of these methods in relation to these parameters. However, they are all positive, and their modules are very significant. Characteristically, for each of the complexes considered here, the smallest values of Δ*_f_H*^0^_298_ and Δ*_f_G*^0^_298_ are observed in the case of using the DFT OPBE/TZVP and the largest in the case of using the DFT M06/TZVP. 

The DFT B3PW91/TZVP and DFT M06/TZVP calculated standard thermodynamic parameters of the template synthesis reactions leading to the formation of these complexes (the general scheme of which is shown in Figure 2) as given in Table 4. As can be seen from it, the values of each of these parameters calculated by these two versions of the DFT are quite close to each other. That is, characteristically, for all these reactions, the relations Δ_r_*H*^0^_298_ < 0, Δ_r_*S*^0^_298_ > 0, and Δ_r_*G*^0^_298_ < 0 take place. From this, in turn, it follows that for any of the M = Ni, Cu, Zn considered by us, such reactions are thermodynamically allowed not only under standard conditions with *T* = 298.16 K but also at any other temperature *T*, since according to the Gibbs–Helmholtz equation for an isobaric process Δ_r_*G*(*T*) = Δ_r_*H*^0^ − *T*Δ_r_*S*^0^ the values of Δ_r_*G*(*T*) for these reactions will always be negative. It should be noted, however, that the data presented in Table 4 refer to the gas phase, and therefore, our conclusion regarding the possibility of their implementation also applies to reactions occurring precisely under such conditions. 

According to our data, the ground state of the Ni**L** and Zn**L** chelates in each of the DFT variants used by us is a spin singlet and that of the Cu**L** chelate is a spin doublet, so that all of them belong to the category of low-spin complexes. This is confirmed by the calculation data of the <S**2> parameter, which is equal to 0.0000 in the case of the Ni**L** and Zn**L** complexes and 0.7500 in the case of CuL, which correspond to the spin multiplicities *M_S_* = 1 and *M_S_* = 2, respectively. Wherein, the difference in the energies of structures with a spin multiplicity different from that of the ground state (triplet in the case of Ni**L** and Zn**L**, quartet in the case of Cu**L**) is 132.3, 146.7, and 144.7 kJ/mol (according to DFT B3PW91/TZVP), 148.7, 157.3, and 200.1 kJ/mol (according to DFT B3PW91/TZVP), and 101.6, 120.7, and 124.3 kJ/mol (according to DFT OPBE/TZVP), respectively. As can be seen from these data, the nearest excited state in each of the complexes under study is much higher than the ground state, so that spin cross-over (spin isomerism) is impossible here in principle. That is, interestingly, the largest differences between the energies of the ground and nearest excited states with a different spin multiplicity, as well as for the parameters Δ*_f_H*^0^_298_ and Δ*_f_G*^0^_298_, take place in the case of using the DFT M06/TZVP and the smallest in the case of using the DFT OPBE/TZVP. 

## 3. Calculation Method

When performing calculations, we used a variant of the density functional theory (DFT), which combines the standard extended valence-split basis set TZVP and the most modern hybrid functional M06, described in detail in [11], which, according to its authors, is best suited for calculations of 3*d*-element compounds. For comparison, we also used another version of the DFT, namely, with B3PW91 functional, which is described in detail in [12,13,14] and used by us, in particular, in recently published papers [15,16,17]. The use of this variant of the DFT, in this case, is due to the fact that, according to [12,13,14] and our experience, it allows, as a rule, to obtain the most accurate (i.e., close to experimental) values of the geometric parameters of molecular structures, as well as significantly more accurate values of thermodynamic and other physicochemical parameters compared to other variants of DFT chemistry models. In addition to them, we also carried out calculations of the molecular and electronic structures of the macrocyclic metal chelates by the OPBE/TZVP method, which combines the above TZVP basis and the non-hybrid OPBE functional [18,19], which, according to the data of works [19,20,21,22,23], in the case of *3d* elements gives a fairly accurate ratio of the energy of the high-spin state with respect to the energy of the low-spin state and, at the same time, reliably characterizes the geometric parameters of the molecular structures of the metal complexes observed by us. The calculations were carried out using the Gaussian09 software package [24]. As in our previous articles, in which the above calculation methods [15,16,17] were used, the correspondence of the found stationary points to the energy minima in all cases was proved by calculating the second derivatives of the energy with respect to the atomic coordinates; in this case, all the equilibrium structures corresponding to the minimum points on the potential energy surfaces had only real (and, moreover, always positive) frequency values. From the optimized structures, the structure with the lowest total energy was chosen for further consideration. In accordance with the theory of the structure of the atoms, Ni(II), Cu(II), and Zn(II) which are the complexes under examination, should correspond to 3*d*^8^, 3*d*^9^, and 3*d*^10^ electronic configurations, respectively. In this context, spin multiplicities 1 and 3 in the case of Ni(II) and Zn(II) and spin multiplicities 2 and 4 in the case of Cu(II) are considered for the given central metal ions during the calculation. Among the structures optimized at these multiplicities, the lowest-lying structure was selected. To calculate the parameters of molecular structures with multiplicity greater than 1, we used the unrestricted method (*UM06*, *UB3PW91*, and *UOPBE*). The energetically most favorable structure has always been checked according to the STABLE = OPT procedure; in all cases, the wave functions corresponding to these structures were stable. Natural Bond Orbital (NBO) analysis was carried out, using the NBO 3.1 version in the framework of the Gaussian09 program [24] according to the methodology described in [25]. The standard thermodynamic parameters of a formation (Δ*_f_H*^0^_298_, *S_f_*^0^_298_, and Δ*_f_G*^0^_298_) for the Ni(II), Cu(II), and Zn(II) macrocyclic compounds under study were calculated employing the method [26].

## 4. Conclusions

Interest in metal macrocyclic compounds and prediction of their physicochemical characteristics (including the calculation of molecular and electronic structures using quantum-chemical calculations of various levels) continues to be consistently high on the part of researchers, as evidenced by a number of recent works, in particular, [27,28,29,30,31,32,33,34,35,36,37,38,39,40], and this article is just one of many devoted to the above topics. In summary, we would like to emphasize that the above-mentioned data of quantum-chemical calculations performed using three different variants of density functional theory, namely, B3PW91/TZVP, M06/TZVP, and OPBE/TZVP chemistry models, unambiguously predict the possibility of the existence of macrocyclic metal complexes Ni(II), Cu(II), and Zn(II) with macrocyclic tetradentate ligand, double-deprotonated form 3,10-dithio-6,7,13,14-tetramethyl-1,2,4,5,8,9,11,12-octaazacyclotetradecatetraene-1,5,7,12 (L^2−^) with the ratio M(II): L^2−^ = 1:1, resulting from template synthesis in the ternary systems M(II)–thiocarbohydrazide–diacetyl (M = Ni, Cu, Zn). Wherein, the reactions of template synthesis in each of these three ternary systems are accompanied by a decrease in enthalpy and an increase in the entropy of the reaction system; the latter circumstance is unusual for template processes, which, as a rule, are accompanied by a decrease in this thermodynamic parameter. Based on the values of Δ*_f_H*^0^_298_, *S_f_*^0^_298_, and Δ*_f_G*^0^_298_, it can be expected that the NiL complex will be the most stable among them, the CuL complex will be the least stable, and the ZnL complex will occupy an intermediate position. Remarkably, only the least stable of these complexes is currently known [1]. In this regard, there is every reason to hope that the other two will also be obtained, and at present it is important to confirm the theoretical prediction made in the experiment.

The key structural fragments of these complexes, namely, chelate nodes, two 5-membered and two 6-membered metal chelate rings, are practically coplanar (which is also unconventional for metal chelates containing the listed atomic groups). The values of the key parameters of molecular structures (bond lengths and bond angles) in the compounds under consideration depend little on the nature of M(II) in their composition. Thus, the decisive role in the formation of these structures belongs, as expected, to the macrocyclic ligand itself.

## Figures and Tables

**Table 1:** Key parameters of the molecular structures of Ni(II), Cu(II), and Zn(II) complexes with the double-deprotonated form of the macrocyclic ligand, 3,10-dithio-6,7,13,14-tetramethyl-1,2,4,5,8,9,11,12-octaazacyclotetradecatetraene-1,5,7,12 (**L**^2−^) calculated by B3PW91/TZVP, M06/TZVP, and OPBE/TZVP chemistry models.

Complex	NiL			CuL			ZnL		
	Chemistry Model			Chemistry Model			Chemistry Model		
Structural Parameter	B3PW91/TZVP	M06/TZVP	OPBE/TZVP	B3PW91/TZVP	M06/TZVP	OPBE/TZVP	B3PW91/TZVP	M06/TZVP	OPBE/TZVP
Bond lengths in the MN_4_ chelate node, pm									
(M1N1)	183.9	184.2	183.4	188.9	188.8	189.4	191.0	190.5	191.4
(M1N2)	183.5	184.2	182.6	190.9	191.4	190.9	197.5	198.2	197.7
(M1N3)	183.9	184.2	183.4	188.9	188.8	189.4	191.0	190.5	191.3
(M1N4)	183.5	184.2	182.6	190.9	191.4	190.9	197.5	198.2	197.6
Separate bond lengths outside the MN_4_ chelate node, pm									
(C1S1), (C2S2)	165.9	165.7	165.9	166.0	165.7	166.1	166.0	165.7	166.0
(C1N5), (C2N6)	137.8	138.0	137.7	139.8	140.0	140.0	141.6	141.7	141.8
(N2N6), (N4N5)	135.6	135.7	135.1	134.9	134.9	134.2	134.3	134.3	133.8
(C4C5), (C3C6)	145.7	146.0	145.1	147.5	147.7	146.9	148.8	148.8	148.2
(C5N7), (C6N8)	130.1	129.5	131.2	130.4	129.8	131.6	130.7	130.0	132.0
(C6C8), (C5C9)	150.6	150.1	150.6	150.8	150.3	150.9	151.0	150.4	151.1
(C4C10), (C7C8)	149.9	149.4	149.9	150.0	149.5	150.1	150.1	150.0	150.2
Bond angles in the MN_4_ chelate node, deg									
(N1M1N2)	94.1	94.1	94.1	94.7	94.7	94.7	95.1	95.2	95.0
(N2M1N3)	85.9	85.9	85.9	85.3	85.3	85.3	84.9	84.8	85.0
(N3M1N4)	94.1	94.1	94.1	94.7	94.7	94.7	95.1	95.2	95.0
(N4M1N1)	85.9	85.9	85.9	85.3	85.3	85.3	84.9	84.8	85.0
Bond angles sum (BAS)	360.0	360.0	360.0	360.0	360.0	360.0	360.0	360.0	360.0
Non-bond angles in the N_4_ grouping, deg									
(N1N2N3)	90.1	90.0	90.2	89.4	89.2	89.5	88.1	87.7	88.2
(N2N3N4)	89.9	90.0	89.8	90.6	90.8	90.5	91.9	92.3	91.8
(N3N4N1)	90.1	90.0	90.2	89.4	89.2	89.5	88.1	87.7	88.2
(N4N1N2)	89.9	90.0	89.8	90.6	90.8	90.5	91.9	92.3	91.8
Non-bond angles sum (NBAS)	360.0	360.0	360.0	360.0	360.0	360.0	360.0	360.0	360.0
Bond angles in the 5-numbered (M1N4N5C1N1) chelate ring, deg									
(M1N4N5)	110.5	110.3	110.9	109.0	108.9	109.1	107.9	107.6	107.8
(N4N5C1)	119.1	119.2	119.3	120.5	120.5	120.9	121.1	121.0	121.6
(N5C1N1)	109.7	109.8	109.1	111.1	111.1	110.7	111.9	112.0	111.6
(C1N1M1)	114.8	114.8	114.8	114.1	114.2	114.0	114.2	114.5	114.0
(N1M1N4)	85.9	85.9	85.9	85.3	85.3	85.3	84.9	84.8	85.0
Bond angles sum (VAS^51^)	540.0	540.0	540.0	540.0	540.0	540.0	540.0	540.0	540.0
Bond angles in the 5-numbered (M1N2N6C2N3) chelate ring, deg									
(M1N2N6)	110.5	110.3	110.9	109.0	108.9	109.1	107.9	107.6	107.8
(N2N6C2)	119.1	119.2	119.3	120.5	120.5	120.9	121.1	121.0	121.6
(N6C2N3)	109.7	109.8	109.1	111.1	111.1	110.7	111.9	112.0	111.6
(C2N3M1)	114.8	114.8	114.8	114.1	114.2	114.0	114.2	114.5	114.0
(N3M1N2)	85.9	85.9	85.9	85.3	85.3	85.3	84.9	84.8	85.0
Bond angles sum (VAS^52^)	540.0	540.0	540.0	540.0	540.0	540.0	540.0	540.0	540.0
Bond angles in the 6-numbered (M1N1N7C5C4N2) chelate ring, deg									
(M1N1N7)	128.5	128.3	128.8	127.0	126.8	127.1	126.1	125.9	126.6
(N1N7C5)	122.2	122.4	122.3	122.3	122.5	122.4	122.2	122.3	121.7
(N7C5C4)	127.0	127.2	126.8	129.5	129.5	129.6	131.6	131.6	132.1
(C5C4N2)	120.3	120.4	119.9	120.4	120.5	120.0	120.0	120.1	119.4
(C4N2M1)	127.9	127.6	128.1	126.1	126.0	126.2	125.0	124.4	125.2
(N2M1N1)	94.1	94.1	94.1	94.7	94.7	94.7	95.1	95.2	95.0
Bond angles sum (VAS^61^)	720.0	720.0	720.0	720.0	720.0	720.0	720.0	719.5	720.0
Bond angles in the 6-numbered (M1N3N8C6C3N4) chelate ring, deg									
(M1N3N8)	128.5	128.3	128.8	127.0	126.8	127.1	126.1	125.9	126.6
(N3N8C6)	122.2	122.4	122.3	122.3	122.5	122.4	122.2	122.3	121.7
(N8C6C3)	127.0	127.2	126.8	129.5	129.5	129.6	131.6	131.6	132.1
(C6C3N4)	120.3	120.4	119.9	120.4	120.5	120.0	120.0	120.1	119.4
(C3N4M1)	127.9	127.6	128.1	126.1	126.0	126.2	125.0	124.4	125.2
(N4M1N3)	94.1	94.1	94.1	94.7	94.7	94.7	95.1	95.2	95.0
Bond angles sum (VAS^62^)	720.0	720.0	720.0	720.0	720.0	720.0	720.0	719.5	720.0
Bond angles outside chelate rings, deg									
(N1C1S1), (N3C2S2)	130.6	130.6	130.9	130.7	130.6	131.1	130.8	130.7	131.4
(N5N4C3), (N6N2C4)	121.6	122.1	121.0	124.8	125.2	124.6	127.2	127.6	127.1
(N4C3C7), (N2C4C10)	118.8	118.7	119.1	118.6	118.5	118.9	118.8	119.3	119.0
(C3C6C8), (C4C5C9)	118.8	118.4	119.6	117.3	117.0	117.9	116.1	115.6	116.6
(C8C6N8), (C9C5N7)	114.1	114.4	113.6	113.2	113.5	112.4	112.2	112.8	111.3
(C6C3C7), (C5C4C10)	120.9	120.8	121.0	121.0	121.0	121.1	121.3	120.6	121.5

**Table 2:** Key data of NBO analysis for the Ni**L**, Cu**L**, and Zn**L** complexes in the ground state according to DFT B3PW91/TZVP, DFT M06/TZVP, and DFT OPBE/TZVP chemistry models.

Complex	Chemistry Model	The Charges on the Atoms, in Electron Charge Units (ē)					<S**2>
		M1	N1	N2	N3	N4	
NiL	B3PW91/TZVP	+0.379	−0.316	−0.187	−0.316	−0.187	0.0000
	M06/TZVP	+0.382	−0.335	−0.198	−0.335	−0.198	0.0000
	OPBE/TZVP	+0.314	−0.265	−0.173	−0.265	−0.173	0.0000
CuL	B3PW91/TZVP	+0.729	−0.411	−0.257	−0.411	−0.257	0.7500
	M06/TZVP	+0.711	−0.425	−0.262	−0.425	−0.262	0.7500
	OPBE/TZVP	+0.672	−0.364	−0.245	−0.364	−0.245	0.7500
ZnL	B3PW91/TZVP	+1.088	−0.508	−0.321	−0.509	−0.321	0.0000
	M06/TZVP	+1.071	−0.524	−0.326	−0.524	−0.326	0.0000
	OPBE/TZVP	+1.085	−0.475	−0.318	−0.475	−0.318	0.0000

**Table 3:** Standard thermodynamic parameters of formation for Ni**L**, Cu**L**, and Zn**L** complexes calculated by B3PW91/TZVP, M06/TZVP, and OPBE/TZVP chemistry models.

Complex	Chemistry Model	*Δ_f_H*^0^_298_, kJ/mol	*S_f_*^0^_298_, J/mol K	*Δ_f_G*^0^_298_, kJ/mol
NiL	B3PW91/TZVP	769.1	745.5	966.6
	M06/TZVP	891.0	742.7	1089.4
	OPBE/TZVP	550.1	753.4	745.3
CuL	B3PW91/TZVP	916.4	748.5	1114.1
	M06/TZVP	1052.2	753.7	1248.3
	OPBE/TZVP	753.3	760.5	947.4
ZnL	B3PW91/TZVP	834.8	772.5	1027.9
	M06/TZVP	983.9	766.1	1178.9
	OPBE/TZVP	669.8	771.5	863.2

**Table 4:** Standard thermodynamic parameters of template synthesis reactions of NiL, CuL, and ZnL complexes in gaseous phase calculated by B3PW91/TZVP and M06/TZVP chemistry models.

Complex	Chemistry Model	Δ*_r_H*^0^_298_, kJ	Δ*_r_S_r_*^0^_298_, J/K	Δ*_r_G*^0^_298_, kJ
NiL	B3PW91/TZVP	−411.6	46.6	−425.5
	M06/TZVP	−393.1	65.6	−412.7
CuL	B3PW91/TZVP	−204.7	64.1	−223.8
	M06/TZVP	−191.2	91.2	−218.4
ZnL	B3PW91/TZVP	−77.0	80.5	−101.0
	M06/TZVP	−66.7	96.0	−95.3

## Data Availability

No unpublished data were created or analyzed in this article.

## Supplementary Materials

The following supporting information can be downloaded at: https://www.mdpi.com/article/10.3390/molecules28114383/s1, NBO Analysis Data of ML complexes.
