# Supplementary material for: Molecular and Electronic Structures of Macrocyclic Compounds Formed at Template Synthesis in the M(II)—Thiocarbohydrazide—Diacetyl Triple Systems: A Quantum-Chemical Analysis by DFT Methods
Source: Molecules. 2023 May 27;28(11):4383. doi: 10.3390/molecules28114383 (PMC10254634; doi:10.3390/molecules28114383)
Supplement: Supplementary file 1 [file molecules-28-04383-s001.zip › molecules-2412359-supplementary.pdf]

NBO Analysis Data of NiL complex (L^{2-} is double deprotonated form of 3,10-dithio-6,7,13,14-tetramethyl-1,2,4,5,8,9,11,12-octaazacyclotetradecatetraene-1,5,7,12)

DFT B3PW91/TZVP level

ΔE(multipl.=1) = 0.0 кДж/моль
ΔE(multipl.=3) = 132.3 кДж/моль

Alpha occupied eigenvalues (highest) = -5.6697477 eV
Alpha virtual eigenvalues (lowest) = -3.091056 eV

<S**2> = 0.0000

Summary of Natural Population Analysis:

Atom	No	Natural Charge	Natural Population Core	Valence	Rydberg	Total
Ni	1	0.37942	17.99148	9.61737	0.01174	27.62058
N	2	-0.31645	1.99918	5.28260	0.03466	7.31645
N	3	-0.18726	1.99907	5.15745	0.03074	7.18726
N	4	-0.31649	1.99918	5.28264	0.03466	7.31649
N	5	-0.18726	1.99907	5.15745	0.03075	7.18726
N	6	-0.39196	1.99939	5.37012	0.02245	7.39196
N	7	-0.39199	1.99939	5.37015	0.02245	7.39199
H	8	0.41893	0.00000	0.57800	0.00307	0.58107
H	9	0.41895	0.00000	0.57798	0.00307	0.58105
C	10	0.18589	1.99941	3.78055	0.03414	5.81411
C	11	0.18591	1.99941	3.78054	0.03414	5.81409
S	12	-0.14926	9.99902	6.12918	0.02106	16.14926
S	13	-0.14929	9.99902	6.12921	0.02106	16.14929
N	14	-0.14289	1.99930	5.12059	0.02300	7.14289
N	15	-0.14286	1.99930	5.12056	0.02300	7.14286
C	16	0.20388	1.99918	3.77647	0.02047	5.79612
C	17	0.20390	1.99918	3.77645	0.02047	5.79610
C	18	0.11132	1.99921	3.86598	0.02350	5.88868
C	19	0.11131	1.99921	3.86598	0.02350	5.88869
C	20	-0.68313	1.99941	4.67541	0.00831	6.68313
H	21	0.23468	0.00000	0.76354	0.00178	0.76532
H	22	0.23466	0.00000	0.76356	0.00178	0.76534
H	23	0.25018	0.00000	0.74868	0.00114	0.74982
C	24	-0.63903	1.99942	4.63163	0.00799	6.63903
H	25	0.21362	0.00000	0.78501	0.00137	0.78638
H	26	0.21362	0.00000	0.78501	0.00137	0.78638
H	27	0.25351	0.00000	0.74518	0.00131	0.74649
C	28	-0.63904	1.99942	4.63163	0.00799	6.63904
H	29	0.21362	0.00000	0.78501	0.00137	0.78638
H	30	0.25351	0.00000	0.74517	0.00131	0.74649
H	31	0.21362	0.00000	0.78501	0.00137	0.78638
C	32	-0.68312	1.99941	4.67540	0.00831	6.68312
H	33	0.23468	0.00000	0.76354	0.00178	0.76532
H	34	0.23466	0.00000	0.76356	0.00178	0.76534
H	35	0.25018	0.00000	0.74868	0.00114	0.74982
* Total *		-0.00000	73.97666	115.53529	0.48806	190.00000

M06/TZVP level

ΔE(multipl.=1) = 0.0 кДж/моль
ΔE(multipl.=3) = 148.7 кДж/моль

Alpha occupied eigenvalues (highest) = -5.888244 eV
Alpha virtual eigenvalues (lowest) = -2.8456218 eV

<S**2> = 0.0000

Summary of Natural Population Analysis:

Atom	No	Natural Charge	Natural Population Core	Valence	Rydberg	Total
Ni	1	0.38236	17.99170	9.61426	0.01167	27.61764
N	2	-0.33535	1.99918	5.30298	0.03319	7.33535
N	3	-0.19794	1.99906	5.16945	0.02943	7.19794
N	4	-0.33539	1.99918	5.30303	0.03318	7.33539
N	5	-0.19794	1.99906	5.16945	0.02943	7.19794
N	6	-0.40747	1.99939	5.38632	0.02177	7.40747
N	7	-0.40750	1.99939	5.38634	0.02177	7.40750
H	8	0.41423	0.00000	0.58283	0.00294	0.58577
H	9	0.41425	0.00000	0.58281	0.00294	0.58575
C	10	0.21038	1.99941	3.75606	0.03415	5.78962
C	11	0.21038	1.99941	3.75605	0.03415	5.78962
S	12	-0.14902	9.99903	6.12940	0.02058	16.14902
S	13	-0.14904	9.99903	6.12943	0.02058	16.14904
N	14	-0.14646	1.99928	5.12375	0.02342	7.14646
N	15	-0.14643	1.99929	5.12372	0.02342	7.14643
C	16	0.22884	1.99917	3.75193	0.02006	5.77116
C	17	0.22887	1.99917	3.75191	0.02006	5.77113
C	18	0.12195	1.99918	3.85654	0.02233	5.87805
C	19	0.12195	1.99918	3.85654	0.02233	5.87805
C	20	-0.67040	1.99939	4.66275	0.00826	6.67040
H	21	0.23019	0.00000	0.76803	0.00178	0.76981
H	22	0.23017	0.00000	0.76805	0.00178	0.76983
H	23	0.24303	0.00000	0.75594	0.00103	0.75697
C	24	-0.62323	1.99940	4.61605	0.00778	6.62323
H	25	0.20675	0.00000	0.79185	0.00140	0.79325
H	26	0.20676	0.00000	0.79185	0.00140	0.79324
H	27	0.24640	0.00000	0.75235	0.00125	0.75360
C	28	-0.62324	1.99940	4.61606	0.00778	6.62324
H	29	0.20676	0.00000	0.79184	0.00140	0.79324
H	30	0.24641	0.00000	0.75234	0.00125	0.75359
H	31	0.20676	0.00000	0.79185	0.00140	0.79324
C	32	-0.67040	1.99939	4.66275	0.00826	6.67040
H	33	0.23019	0.00000	0.76803	0.00178	0.76981
H	34	0.23017	0.00000	0.76805	0.00178	0.76983
H	35	0.24303	0.00000	0.75594	0.00103	0.75697
* Total *		-0.00000	73.97668	115.54653	0.47679	190.00000

OPBE/TZVP level

ΔE(multipl.=1) = 0.0 кДж/моль
ΔE(multipl.=3) = 101.6 кДж/моль

Alpha occupied eigenvalues (highest) = -4.7005275 eV
Alpha virtual eigenvalues (lowest) = -3.4847847 eV

<S**2> = 0.0000

Summary of Natural Population Analysis:

Atom	No	Natural Charge	Natural Population Core	Valence	Rydberg	Total
Ni	1	0.31367	17.99128	9.68066	0.01439	27.68633
N	2	-0.26538	1.99921	5.23324	0.03293	7.26538
N	3	-0.17255	1.99910	5.14287	0.03058	7.17255
N	4	-0.26545	1.99921	5.23331	0.03292	7.26545
N	5	-0.17254	1.99910	5.14286	0.03058	7.17254
N	6	-0.36457	1.99940	5.34487	0.02031	7.36457
N	7	-0.36459	1.99940	5.34489	0.02031	7.36459
H	8	0.42115	0.00000	0.57555	0.00331	0.57885
H	9	0.42117	0.00000	0.57552	0.00331	0.57883
C	10	0.13375	1.99942	3.83568	0.03116	5.86625
C	11	0.13379	1.99942	3.83563	0.03116	5.86621
S	12	-0.12198	9.99901	6.10353	0.01944	16.12198
S	13	-0.12206	9.99901	6.10361	0.01943	16.12206
N	14	-0.12851	1.99933	5.10737	0.02181	7.12851
N	15	-0.12847	1.99933	5.10734	0.02181	7.12847
C	16	0.16403	1.99919	3.81760	0.01918	5.83597
C	17	0.16404	1.99919	3.81759	0.01918	5.83596
C	18	0.09909	1.99921	3.88012	0.02158	5.90091
C	19	0.09909	1.99921	3.88012	0.02158	5.90091
C	20	-0.70424	1.99940	4.69778	0.00705	6.70424
H	21	0.24029	0.00000	0.75789	0.00182	0.75971
H	22	0.24028	0.00000	0.75790	0.00182	0.75972
H	23	0.25924	0.00000	0.73956	0.00121	0.74076
C	24	-0.66552	1.99942	4.65944	0.00667	6.66552
H	25	0.22384	0.00000	0.77477	0.00139	0.77616
H	26	0.22384	0.00000	0.77477	0.00139	0.77616
H	27	0.26044	0.00000	0.73814	0.00143	0.73956
C	28	-0.66553	1.99942	4.65944	0.00667	6.66553
H	29	0.22385	0.00000	0.77477	0.00139	0.77615
H	30	0.26044	0.00000	0.73813	0.00143	0.73956
H	31	0.22384	0.00000	0.77477	0.00139	0.77616
C	32	-0.70423	1.99940	4.69777	0.00705	6.70423
H	33	0.24028	0.00000	0.75789	0.00182	0.75972
H	34	0.24027	0.00000	0.75791	0.00182	0.75973
H	35	0.25922	0.00000	0.73957	0.00121	0.74078
* Total *		-0.00000	73.97666	115.56284	0.46049	190.00000

NBO Analysis Data of CuL complex (L^{2-} is double deprotonated form of 3,10-dithio-6,7,13,14-tetramethyl-1,2,4,5,8,9,11,12-octaazacyclotetradecatetraene-1,5,7,12)

DFT B3PW91/TZVP level

ΔE(multipl.=2) = 0.0 кДж/моль
ΔE(multipl.=4) = 144.7 кДж/моль

Alpha occupied eigenvalues (highest) = -5.8327356 eV
Alpha virtual eigenvalues (lowest) = -3.0589482 eV
Beta occupied eigenvalues (highest) = -5.7891996 eV
Beta virtual eigenvalues (lowest) = -3.0439827 eV

<S**2> = 0.7500

Summary of Natural Population Analysis:

Atom	No	Natural Charge	Natural Population			
			Core	Valence	Rydberg	Total
Cu	1	0.72967	17.99418	10.26765	0.00850	28.27033
N	2	-0.41057	1.99926	5.37391	0.03740	7.41057
N	3	-0.25679	1.99917	5.22545	0.03217	7.25679
N	4	-0.41057	1.99926	5.37391	0.03740	7.41057
N	5	-0.25679	1.99917	5.22545	0.03217	7.25679
N	6	-0.39158	1.99942	5.36907	0.02309	7.39158
N	7	-0.39158	1.99942	5.36907	0.02309	7.39158
H	8	0.41419	0.00000	0.58254	0.00327	0.58581
H	9	0.41419	0.00000	0.58254	0.00327	0.58581
C	10	0.18775	1.99943	3.78004	0.03278	5.81225
C	11	0.18775	1.99943	3.78004	0.03278	5.81225
S	12	-0.15300	9.99902	6.13314	0.02085	16.15300
S	13	-0.15300	9.99902	6.13314	0.02085	16.15300
N	14	-0.15319	1.99930	5.12955	0.02434	7.15319
N	15	-0.15319	1.99930	5.12955	0.02434	7.15319
C	16	0.20539	1.99919	3.77425	0.02117	5.79461
C	17	0.20539	1.99919	3.77425	0.02117	5.79461
C	18	0.12140	1.99923	3.85582	0.02355	5.87860
C	19	0.12140	1.99923	3.85582	0.02355	5.87860
C	20	-0.68618	1.99941	4.67831	0.00846	6.68618
H	21	0.23461	0.00000	0.76361	0.00178	0.76539
H	22	0.23453	0.00000	0.76369	0.00178	0.76547
H	23	0.24880	0.00000	0.75001	0.00119	0.75120
C	24	-0.63951	1.99942	4.63209	0.00801	6.63951
H	25	0.21294	0.00000	0.78569	0.00137	0.78706
H	26	0.21294	0.00000	0.78569	0.00137	0.78706
H	27	0.25345	0.00000	0.74519	0.00136	0.74655
C	28	-0.63951	1.99942	4.63209	0.00801	6.63951
H	29	0.21294	0.00000	0.78569	0.00137	0.78706
H	30	0.25345	0.00000	0.74519	0.00136	0.74655
H	31	0.21294	0.00000	0.78569	0.00137	0.78706
C	32	-0.68618	1.99941	4.67831	0.00846	6.68618
H	33	0.23461	0.00000	0.76361	0.00178	0.76539
H	34	0.23453	0.00000	0.76369	0.00178	0.76547
H	35	0.24880	0.00000	0.75001	0.00119	0.75120
* Total *		-0.00000	73.97986	116.52379	0.49635	191.00000

M06/TZVP level

ΔE(multipl.=2) = 0.0 кДж/моль
ΔE(multipl.=4) = 200.1 кДж/моль

Alpha occupied eigenvalues (highest) = -6.0000771 eV
Alpha virtual eigenvalues (lowest) = -2.8067115 eV
Beta occupied eigenvalues (highest) = -5.9862 eV
Beta virtual eigenvalues (lowest) = -2.7925623 eV

<S**2> = 0.7500

Summary of Natural Population Analysis:

Atom	No	Natural Charge	Natural Population: Core	Valence	Rydberg	Total
Cu	1	0.71114	17.99425	10.28591	0.00871	28.28886
N	2	-0.42463	1.99925	5.38941	0.03598	7.42463
N	3	-0.26202	1.99915	5.23179	0.03108	7.26202
N	4	-0.42463	1.99925	5.38941	0.03598	7.42463
N	5	-0.26202	1.99915	5.23179	0.03108	7.26202
N	6	-0.40705	1.99942	5.38522	0.02240	7.40705
N	7	-0.40705	1.99942	5.38522	0.02240	7.40705
H	8	0.40930	0.00000	0.58754	0.00316	0.59070
H	9	0.40930	0.00000	0.58754	0.00316	0.59070
C	10	0.21228	1.99942	3.75534	0.03296	5.78772
C	11	0.21228	1.99942	3.75534	0.03296	5.78772
S	12	-0.15245	9.99903	6.13310	0.02032	16.15245
S	13	-0.15245	9.99903	6.13310	0.02032	16.15245
N	14	-0.15706	1.99928	5.13287	0.02491	7.15706
N	15	-0.15706	1.99928	5.13287	0.02491	7.15706
C	16	0.22830	1.99917	3.75172	0.02081	5.77170
C	17	0.22830	1.99917	3.75172	0.02081	5.77170
C	18	0.13430	1.99920	3.84415	0.02235	5.86570
C	19	0.13430	1.99920	3.84415	0.02235	5.86570
C	20	-0.67328	1.99939	4.66545	0.00844	6.67328
H	21	0.22990	0.00000	0.76832	0.00178	0.77010
H	22	0.22989	0.00000	0.76833	0.00178	0.77011
H	23	0.24171	0.00000	0.75722	0.00107	0.75829
C	24	-0.62377	1.99940	4.61659	0.00778	6.62377
H	25	0.20633	0.00000	0.79228	0.00139	0.79367
H	26	0.20633	0.00000	0.79228	0.00139	0.79367
H	27	0.24635	0.00000	0.75236	0.00129	0.75365
C	28	-0.62377	1.99940	4.61659	0.00778	6.62377
H	29	0.20633	0.00000	0.79228	0.00139	0.79367
H	30	0.24635	0.00000	0.75236	0.00129	0.75365
H	31	0.20633	0.00000	0.79228	0.00139	0.79367
C	32	-0.67328	1.99939	4.66545	0.00844	6.67328
H	33	0.22992	0.00000	0.76830	0.00178	0.77008
H	34	0.22988	0.00000	0.76834	0.00178	0.77012
H	35	0.24171	0.00000	0.75722	0.00107	0.75829
* Total *		-0.00000	73.97968	116.53380	0.48652	191.00000

OPBE/TZVP level

ΔE(multipl.=2) = 0.0 кДж/моль
ΔE(multipl.=4) = 120.7 кДж/моль

Alpha occupied eigenvalues (highest) = -4.1971425 eV
Alpha virtual eigenvalues (lowest) = -3.4787985 eV
Beta occupied eigenvalues (highest) = -4.9995654 eV
Beta virtual eigenvalues (lowest) = -3.4499559 eV

<S**2> = 0.7500

Summary of Natural Population Analysis:

Atom	No	Natural Charge	Natural Population			
			Core	Valence	Rydberg	Total
Cu	1	0.67173	17.99426	10.32433	0.00967	28.32827
N	2	-0.36379	1.99929	5.32896	0.03554	7.36379
N	3	-0.24518	1.99921	5.21408	0.03190	7.24518
N	4	-0.36379	1.99929	5.32896	0.03554	7.36379
N	5	-0.24519	1.99921	5.21409	0.03190	7.24519
N	6	-0.36106	1.99943	5.34078	0.02085	7.36106
N	7	-0.36106	1.99943	5.34078	0.02085	7.36106
H	8	0.41489	0.00000	0.58153	0.00358	0.58511
H	9	0.41489	0.00000	0.58153	0.00358	0.58511
C	10	0.13772	1.99944	3.83311	0.02973	5.86228
C	11	0.13772	1.99944	3.83311	0.02973	5.86228
S	12	-0.12675	9.99901	6.10849	0.01924	16.12675
S	13	-0.12675	9.99901	6.10849	0.01924	16.12675
N	14	-0.13880	1.99933	5.11639	0.02308	7.13880
N	15	-0.13880	1.99933	5.11639	0.02308	7.13880
C	16	0.16882	1.99920	3.81221	0.01978	5.83118
C	17	0.16882	1.99920	3.81221	0.01978	5.83118
C	18	0.10783	1.99924	3.87124	0.02169	5.89217
C	19	0.10783	1.99924	3.87124	0.02169	5.89217
C	20	-0.70753	1.99941	4.70093	0.00719	6.70753
H	21	0.24026	0.00000	0.75794	0.00180	0.75974
H	22	0.24026	0.00000	0.75792	0.00181	0.75974
H	23	0.25727	0.00000	0.74144	0.00129	0.74273
C	24	-0.66621	1.99942	4.66011	0.00668	6.66621
H	25	0.22305	0.00000	0.77556	0.00140	0.77695
H	26	0.22301	0.00000	0.77559	0.00140	0.77699
H	27	0.26034	0.00000	0.73815	0.00150	0.73966
C	28	-0.66621	1.99942	4.66011	0.00668	6.66621
H	29	0.22301	0.00000	0.77559	0.00140	0.77699
H	30	0.26034	0.00000	0.73815	0.00150	0.73966
H	31	0.22305	0.00000	0.77555	0.00140	0.77695
C	32	-0.70753	1.99941	4.70093	0.00719	6.70753
H	33	0.24025	0.00000	0.75795	0.00180	0.75975
H	34	0.24027	0.00000	0.75792	0.00181	0.75973
H	35	0.25727	0.00000	0.74144	0.00129	0.74273
* Total *		-0.00000	73.98022	116.55321	0.46658	191.00000

NBO Analysis Data of ZnL complex (L^{2-} is double deprotonated form of 3,10-dithio-6,7,13,14-tetramethyl-1,2,4,5,8,9,11,12-octaazacyclotetradecatetraene-1,5,7,12)

DFT B3PW91/TZVP level

ΔE(multipl.=1) = 0.0 кДж/моль
ΔE(multipl.=3) = 146.7 кДж/моль

Alpha occupied eigenvalues (highest) = -5.8623945 eV
Alpha virtual eigenvalues (lowest) = -3.0096981 eV

<S**2> = 0.0000

Summary of Natural Population Analysis:

Atom	No	Natural Charge	Natural Population Core	Valence	Rydberg	Total
Zn	1	1.08887	17.99585	10.90968	0.00560	28.91113
N	2	-0.50842	1.99930	5.47319	0.03594	7.50842
N	3	-0.32125	1.99925	5.29207	0.02992	7.32125
N	4	-0.50859	1.99930	5.47335	0.03594	7.50859
N	5	-0.32140	1.99925	5.29221	0.02993	7.32140
N	6	-0.39133	1.99944	5.36821	0.02368	7.39133
N	7	-0.39145	1.99944	5.36835	0.02366	7.39145
H	8	0.41099	0.00000	0.58556	0.00345	0.58901
H	9	0.41097	0.00000	0.58558	0.00345	0.58903
C	10	0.18945	1.99942	3.77739	0.03373	5.81055
C	11	0.18957	1.99942	3.77727	0.03374	5.81043
S	12	-0.15278	9.99901	6.13292	0.02085	16.15278
S	13	-0.15288	9.99901	6.13303	0.02084	16.15288
N	14	-0.17131	1.99931	5.14692	0.02508	7.17131
N	15	-0.17125	1.99931	5.14687	0.02507	7.17125
C	16	0.20292	1.99919	3.77624	0.02165	5.79708
C	17	0.20297	1.99919	3.77619	0.02166	5.79703
C	18	0.13212	1.99924	3.84504	0.02360	5.86788
C	19	0.13204	1.99924	3.84513	0.02359	5.86796
C	20	-0.68905	1.99941	4.68118	0.00846	6.68905
H	21	0.23419	0.00000	0.76404	0.00177	0.76581
H	22	0.23416	0.00000	0.76407	0.00177	0.76584
H	23	0.24757	0.00000	0.75120	0.00122	0.75243
C	24	-0.63989	1.99942	4.63243	0.00804	6.63989
H	25	0.21231	0.00000	0.78632	0.00137	0.78769
H	26	0.21232	0.00000	0.78632	0.00137	0.78768
H	27	0.25377	0.00000	0.74485	0.00139	0.74623
C	28	-0.63991	1.99942	4.63246	0.00804	6.63991
H	29	0.21231	0.00000	0.78632	0.00137	0.78769
H	30	0.25377	0.00000	0.74484	0.00139	0.74623
H	31	0.21231	0.00000	0.78633	0.00137	0.78769
C	32	-0.68908	1.99941	4.68122	0.00845	6.68908
H	33	0.23420	0.00000	0.76403	0.00177	0.76580
H	34	0.23418	0.00000	0.76405	0.00177	0.76582
H	35	0.24758	0.00000	0.75120	0.00122	0.75242
* Total *		-0.00000	73.98183	117.52606	0.49211	192.00000

M06/TZVP level

ΔE(multipl.=1) = 0.0 кДж/моль
ΔE(multipl.=3) = 157.3 кДж/моль

Alpha occupied eigenvalues (highest) = -6.0585786 eV
Alpha virtual eigenvalues (lowest) = -2.7356934 eV

<S**2> = 0.0000

Summary of Natural Population Analysis:

			Natural Population			
Atom	No	Natural Charge	Core	Valence	Rydberg	Total
Zn	1	1.07174	17.99587	10.92718	0.00521	28.92826
N	2	-0.52395	1.99928	5.49017	0.03449	7.52395
N	3	-0.32612	1.99923	5.29802	0.02887	7.32612
N	4	-0.52395	1.99928	5.49017	0.03449	7.52395
N	5	-0.32612	1.99923	5.29802	0.02887	7.32612
N	6	-0.40734	1.99944	5.38508	0.02282	7.40734
N	7	-0.40733	1.99944	5.38508	0.02282	7.40733
H	8	0.40529	0.00000	0.59135	0.00336	0.59471
H	9	0.40529	0.00000	0.59135	0.00336	0.59471
C	10	0.21517	1.99942	3.75133	0.03409	5.78483
C	11	0.21517	1.99942	3.75133	0.03409	5.78483
S	12	-0.15410	9.99902	6.13478	0.02030	16.15410
S	13	-0.15410	9.99902	6.13478	0.02030	16.15410
N	14	-0.17554	1.99929	5.15052	0.02574	7.17554
N	15	-0.17554	1.99929	5.15051	0.02574	7.17554
C	16	0.22636	1.99917	3.75322	0.02125	5.77364
C	17	0.22636	1.99917	3.75322	0.02125	5.77364
C	18	0.14651	1.99921	3.83180	0.02249	5.85349
C	19	0.14651	1.99921	3.83180	0.02249	5.85349
C	20	-0.67488	1.99939	4.66694	0.00855	6.67488
H	21	0.23513	0.00000	0.76328	0.00159	0.76487
H	22	0.22333	0.00000	0.77489	0.00178	0.77667
H	23	0.23981	0.00000	0.75905	0.00114	0.76019
C	24	-0.62345	1.99940	4.61626	0.00779	6.62345
H	25	0.20485	0.00000	0.79380	0.00135	0.79515
H	26	0.20665	0.00000	0.79193	0.00142	0.79335
H	27	0.24643	0.00000	0.75226	0.00131	0.75357
C	28	-0.62345	1.99940	4.61626	0.00779	6.62345
H	29	0.20665	0.00000	0.79194	0.00142	0.79335
H	30	0.24643	0.00000	0.75226	0.00131	0.75357
H	31	0.20485	0.00000	0.79380	0.00135	0.79515
C	32	-0.67489	1.99939	4.66695	0.00855	6.67489
H	33	0.23513	0.00000	0.76328	0.00159	0.76487
H	34	0.22333	0.00000	0.77488	0.00178	0.77667
H	35	0.23981	0.00000	0.75905	0.00114	0.76019
* Total *		0.00000	73.98158	117.53656	0.48186	192.00000

OPBE/TZVP level

ΔE(multipl.=1) = 0.0 кДж/моль
ΔE(multipl.=3) = 124.23 кДж/моль

Alpha occupied eigenvalues (highest) = -5.0090889 eV
Alpha virtual eigenvalues (lowest) = -3.4390719 eV

<S**2> = 0.0000

Summary of Natural Population Analysis:

Atom	No	Natural Charge	Natural Population Core	Valence	Rydberg	Total
Zn	1	1.08463	17.99595	10.91273	0.00669	28.91537
N	2	-0.47478	1.99932	5.44154	0.03392	7.47478
N	3	-0.31822	1.99929	5.28956	0.02936	7.31822
N	4	-0.47501	1.99932	5.44176	0.03393	7.47501
N	5	-0.31857	1.99929	5.28990	0.02938	7.31857
N	6	-0.36041	1.99946	5.33959	0.02136	7.36041
N	7	-0.36054	1.99946	5.33974	0.02134	7.36054
H	8	0.41160	0.00000	0.58468	0.00372	0.58840
H	9	0.41156	0.00000	0.58472	0.00372	0.58844
C	10	0.14175	1.99944	3.82821	0.03060	5.85825
C	11	0.14186	1.99944	3.82809	0.03061	5.85814
S	12	-0.12744	9.99900	6.10918	0.01926	16.12744
S	13	-0.12751	9.99900	6.10924	0.01926	16.12751
N	14	-0.16515	1.99935	5.14198	0.02382	7.16515
N	15	-0.16505	1.99935	5.14190	0.02380	7.16505
C	16	0.17062	1.99920	3.81003	0.02014	5.82938
C	17	0.17062	1.99920	3.81002	0.02015	5.82938
C	18	0.11635	1.99926	3.86262	0.02177	5.88365
C	19	0.11618	1.99926	3.86281	0.02176	5.88382
C	20	-0.71116	1.99941	4.70461	0.00714	6.71116
H	21	0.24018	0.00000	0.75803	0.00179	0.75982
H	22	0.24016	0.00000	0.75805	0.00179	0.75984
H	23	0.25625	0.00000	0.74242	0.00133	0.74375
C	24	-0.66668	1.99942	4.66056	0.00670	6.66668
H	25	0.22197	0.00000	0.77664	0.00139	0.77803
H	26	0.22197	0.00000	0.77664	0.00139	0.77803
H	27	0.26111	0.00000	0.73735	0.00154	0.73889
C	28	-0.66672	1.99942	4.66061	0.00670	6.66672
H	29	0.22196	0.00000	0.77665	0.00139	0.77804
H	30	0.26111	0.00000	0.73735	0.00154	0.73889
H	31	0.22196	0.00000	0.77665	0.00139	0.77804
C	32	-0.71122	1.99941	4.70468	0.00714	6.71122
H	33	0.24019	0.00000	0.75802	0.00179	0.75981
H	34	0.24017	0.00000	0.75804	0.00179	0.75983
H	35	0.25626	0.00000	0.74241	0.00133	0.74374
* Total *		-0.00000	73.98228	117.55701	0.46072	192.00000

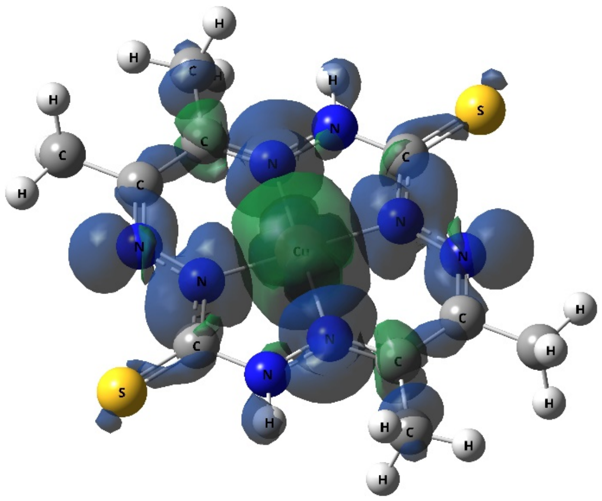

a

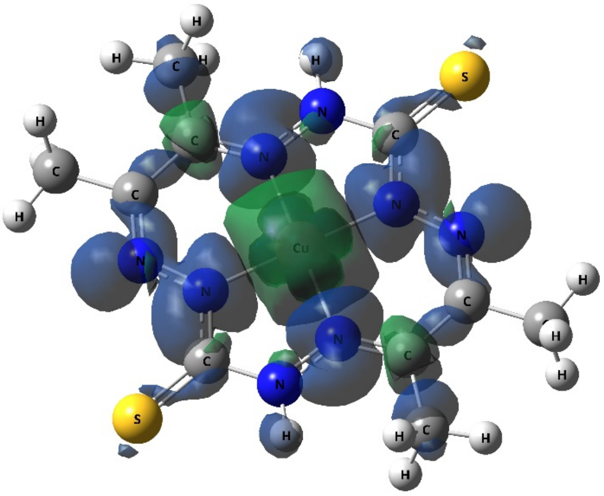

b

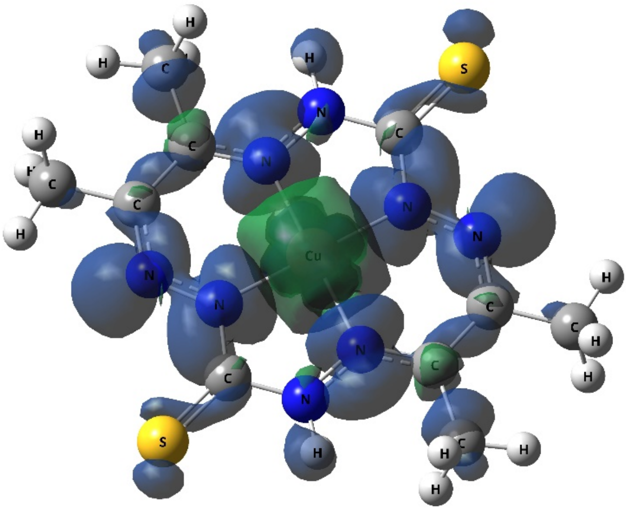

c

Figure S1. Spin density distribution in the Cu**L** complex obtained by DFT B3PW91/ TZVP (*a*), DFT M06/TZVP (*b*) and DFT OPBE/TZVP (*c*) model chemistry.
